# Supplementary material for: Are carbon emissions trading and green financial instruments synergistic? -Comprehensive quantitative research based on content analysis
Source: PLoS One. 2024 Mar 7;19(3):e0298601. doi: 10.1371/journal.pone.0298601 (PMC10919601; doi:10.1371/journal.pone.0298601)
Supplement: S1 Data — (ZIP) [file pone.0298601.s001.zip › Data/地级市Z_F工作报告.zip!/╦─┤¿/┤∩╓▌╩╨/└┤╫╘╣1⁄2╓┌║┼í╛┬φ┐╦╩2╛▌═°í┐_2016.docx]

各位代表：

现在，我代表市人民政府，向大会报告政府工作，请予审查，并请市政协各位委员提出意见。

一、2015年及“十二五”工作回顾

2015年，在省委、省政府和市委的坚强领导下，在市人大、市政协的监督支持下，我们沉着应对多重困难和挑战，深入实施“三大总体战略工程”，扎实开展“五个年”【1】活动，各项工作在攻坚克难中取得新的成绩。全年实现地区生产总值1350.8亿元，增长3.1%；地方一般公共预算收入79.2亿元，增长8.9%；全社会固定资产投资1331.7亿元，增长13.2%；社会消费品零售总额672.5亿元，增长13.5%；城镇居民人均可支配收入23884元，农村居民人均可支配收入10688元，分别增长8.6%和10.1%。

一年来，我们积极应对各种挑战，全力以赴稳增长。认真落实国家和省各项调控政策，制定出台系列稳增长措施，降低工商业用电用气成本2.1亿元，落实减税降费减轻企业负担4.2亿元，实施楼市新政去库存39.4万平方米。集中攻坚25个重大工程项目带动150个重点项目建设，川东北高含硫气田等项目如期建成。主动对接国家战略工程包，争取专项建设基金32.7亿元，位居全省第2。加强银政银企合作，新增贷款投放226亿元。建立PPP项目基金和社会资金引导机制，撬动社会资本近100亿元。积极参与全省市场拓展“三大活动”【2】，成功举办第6届秦巴地区商品交易会，消费对经济增长的贡献率达到53%。

一年来，我们加快转变发展方式，坚定不移调结构。推进工业转型升级，完成技改投资260亿元，竣工投产项目44个，新增规模以上工业企业43户、亿元企业10户，实现规模以上工业增加值289.9亿元。推进服务业集聚发展，秦巴物流园区、红星美凯龙、仁和春天、罗浮新城市广场等重大项目加快建设，服务业增加值增长9.6%。重点旅游景区提档升级，成功举办第五届旅发大会，旅游总收入112亿元。加快现代农业发展，粮食总产实现 “九连增”，新发展特色产业基地10万亩，新改建畜禽标准化规模养殖场176个，新增市级以上龙头企业20家、省级以上标准化农民专合社16个，申报“三品一标”农产品44个。市农科院挂牌成立。促进新业态新模式发展，实施“互联网+”行动计划，建成各类创新平台15家，新培育高新技术企业7家。

一年来，我们着力统筹城乡建设，多措并举强基础。坚持规划引领，提升“四层架构”，城镇化率提高1.48个百分点。强化主城带动，四大城市新区建设和塔沱片区改造有力推进，凤凰山隧道、金南大桥等骨干道路加快建设，城市轨道交通、绕城高速公路前期工作全面启动。“全光网城市”成功建成，“宽带中国”示范城市创建获批。认真落实新环保法，持续推进“五治”工程，整治小街小巷235条，建成停车泊位1万个，新增城镇绿地18万平方米。各县城市新区和产业园区加快发展，17个省级试点镇建设完成投资8.2亿元，新改扩建新村聚居点320个，建成幸福美丽新村200个、村级公共服务中心121个。

一年来，我们扎实推进改革开放，千方百计增活力。市县政府机构改革全面完成。全面取消非行政许可，取消调整行政许可前置条件52项。完成市属国有关停破产企业改革，组建市交通投资集团。“三证合一【3】、一照一码”全面推行，新增市场主体1.98万个。创新设立“税金贷”、“园保贷”，整合组建达州农商银行。农业农村 “6+10”专项改革加快推进，养老保险并轨改革全面启动，教育、卫生等社会事业改革深入推进，机关事业单位工资改革顺利完成。制定实施农民工返乡创业、达商回乡发展政策措施，成功举办首届达商大会。招商引资到位资金655亿元。进出口总额增长13.1%。

一年来，我们大力发展社会事业，真情实意惠民生。十项民生工程、17件民生大事全面完成，各项民生支出229.8亿元，占一般公共预算支出的70.9%。建立产业扶贫信贷基金7.2亿元，争取专项扶贫资金近7亿元，实现脱贫15.1万人。城镇新增就业4.2万人，城镇登记失业率4.0%。五项社会保险参保覆盖人数达到503.4万人次，48.7万城乡低保、4.9万农村特困人员基本生活得到保障。新改扩建中小学校109所，达州嘉祥外国语学校、达州职业技术学院新校区加快建设。分级诊疗制度初步建立，重大传染病防治切实加强。莲花湖演展基地、工青妇活动中心建成投用，“一展一节一赛”【4】成功举办。全民健身活动广泛开展。第三次全国经济普查圆满完成。民族、宗教、统计、档案、地方志、妇女、儿童、老龄、残疾人等工作都取得新的成绩。

一年来，我们切实加强自身建设，持续用力转作风。扎实开展“三严三实”专题教育，行政机关工作作风进一步改进。严格落实党风廉政建设“两个责任”，加强监察、审计工作，依法规范权力行使。认真落实依法治市纲要，推行政府权力清单和责任清单。自觉接受市人大及其常委会法律监督、工作监督和市政协民主监督，人大代表议案及建议意见、政协提案按时办结。深入推进“法律七进”，“六五”普法圆满收官。加强安全生产、食品药品、农产品执法监管，加强社会治安综合整治，有效处置地方金融风险，社会保持和谐稳定。加强国防动员、人民防空、民兵预备役建设和交通战备、双拥优抚等工作，成功创建省级双拥模范城市。

各位代表，随着2015年的过去，“十二五”发展已载入史册。“十二五”时期，世界经济持续深度调整，国内“三期叠加”影响不断加深，我市接连遭受严重自然灾害。面对严峻复杂形势，市委、市政府总揽全局、沉着应对，团结带领全市人民，加快建设川渝陕结合部区域中心城市和川东北经济区核心增长极，在同步全面小康道路上迈出了更加坚实的步伐。

这五年，是我市综合实力大幅提升的五年。地区生产总值由819.2亿元增加到1350.8亿元，年均增长10%。金融机构各项存贷款余额分别突破2000亿元和900亿元，地方一般公共预算收入年均增长20.9%，支持发展的能力显著增强。内需拉动成效明显，全社会固定资产投资累计完成5015亿元，社会消费品零售总额增长1.2倍。县域经济竞相发展，经济总量全部跨入“百亿县”行列，宣汉、大竹被列为全省首批14个工业强县示范县。三次产业结构由23.8：50.0：26.2调整为21.5：48.7：29.8，新的经济增长动力正在加快形成。

这五年，是我市基础设施显著改善的五年。区域综合交通枢纽加速构建。达州机场开通航线达到8条，新机场建设获得批复。巴达铁路建成通车，铁路通车里程达到665公里，达渝城际铁路进入国家规划，市、县火车客站扩能改造竣工投用。公路总里程突破2万公里，达万、达陕、达巴高速公路建成通车，南大梁高速公路即将建成，新改建县乡油（水泥）路2489公里、通村水泥路7971公里，实现县县通高速、乡乡通油（水泥）路目标。渠江流域综合整治扎实推进，6座中型水库相继开工建设，土溪口大型水库前期工作全面完成。农村电网改造、“气化达州”加快实施。智慧城市加快建设，光纤通信、电话、互联网通达90%以上的行政村。

这五年，是我市城乡区域协调发展的五年。新型城镇化进程加快推进，城镇化率达到40.87%。中心城区加速拓展，达县撤县设区、经开区扩区调位顺利完成，建成区面积76平方公里、人口达到90万。5个县城规模不断扩大，“百镇建设行动”培育省级示范城镇17个。农村面貌得到改善，新建高标准农田75万亩，新改扩建新村聚居点1722个、幸福美丽新村249个。成功创建省级森林城市、园林城市，4个县跻身省级绿化模范县行列。累计完成营造林182万亩，森林覆盖率达到42%。县县建成垃圾污水处理设施，“十二五”节能减排目标全面完成。

这五年，是我市改革开放重大突破的五年。制定6大重点领域和22个关键环节的改革框架，农业农村“6+10”专项改革先行先试，简政放权、国资国企、投融资体制、社会建设领域等改革取得重大进展。达州全域纳入成渝经济区规划、川陕革命老区振兴发展规划，并被省委、省政府确定为川东北经济区核心增长极。切实加强开放合作，招商引资累计到位资金2586亿元，新增外贸经营者备案企业247家，进出口总额年均增长24.9%。

这五年，是我市社会建设全面加强的五年。财政用于民生投入921亿元，是“十一五”的1.7倍。城镇新增就业18.5万人。实现城乡居民基本养老保险制度全覆盖，城乡三项医疗保险【5】参保率达97%，人均预期寿命提高到76岁。改造城镇危旧房、棚户区和建设保障性住房8.3万套，完成农村危房改造9.7万户。扶贫脱贫71.8万人，贫困发生率由21.9%下降到9.2%。183.5万人饮水安全问题得到解决。义务教育全面实行“三免一补”，各类教育加快发展。创造科技成果373项，专利申请3500件。文化、体育事业繁荣发展，连续五届成功举办全国新农村文化艺术展演。民主法制建设不断加强，社会治理能力显著提升，和谐稳定局面进一步巩固。

各位代表，五年的开拓奋进，五年的辉煌成就，奠定了达州科学发展、全面转型的坚实基础，开启了达州加速崛起、脱贫奔康的崭新局面。这是省委、省政府和市委正确领导、科学决策的结果，是700万达州人民团结奋斗、顽强拼搏的结果。在此，我代表市人民政府，向全市人民，向人大代表、政协委员，向各民主党派、工商联、无党派代表人士和人民团体，向驻达解放军指战员、武警官兵和政法干警，向所有关心支持达州建设发展的社会各界人士，表示衷心的感谢！

在充分肯定成绩的同时，我们也清醒地认识到，政府工作还存在不少困难和问题。主要是：受宏观经济环境持续低迷、自身结构矛盾突出等因素影响，地区生产总值、规模以上工业增加值未能实现预期；传统产业产能过剩，新兴产业发展不足，增长动力亟待转换，转型发展任重道远；资源、环境问题比较突出；城乡区域发展不平衡，基础设施、公共服务欠账较大，贫困人口较多，改善民生和脱贫攻坚任务艰巨；简政放权、职能转变还有差距，少数政府机关还存在懒政怠政等现象，依法行政的意识和能力还需进一步提高。对此，我们一定高度重视，采取有效措施，切实加以解决。

二、“十三五”时期主要目标任务

“十三五”时期，是我市转型发展的关键时期，也是脱贫奔康的决胜时期。根据中央、省委精神和市委“十三五”规划《建议》，市政府组织编制了《达州市国民经济和社会发展第十三个五年规划纲要（草案）》，提交本次大会一并审议。

“十三五”发展的指导思想是：认真贯彻党的十八大和十八届三中、四中、五中全会和省委十届七次全会精神，坚持“四个全面”战略布局，全面践行“五大”发展理念，坚持“增量促转型、存量调结构、改革强动力、创新激活力、开放增实力”的基本思路，持续推进“三大总体战略工程”，做强“三大功能性支撑”【6】，实施“六大崛起行动计划”【7】，负重爬坡转型，高点切入起跳，后发追赶跨越，确保与全国全省同步全面建成小康社会，奋力走在四川改革发展前列。

今后五年发展的主要目标是：保持高于全省1个百分点的经济增速，到2020年，经济总量、城乡居民人均可支配收入比2010年翻一番，地区生产总值达到2000亿元以上，人民生活水平大幅提高，绝对贫困全面消除。生态文明建设取得显著成效，中心城区空气质量达标率上升5个百分点，森林覆盖率达到43.5%。改革实现重大突破，开放型经济新体制基本形成。公民素质和社会文明程度普遍提升，民主法制建设取得重大进展，法治政府基本建成。通过五年努力，把达州建成川渝陕结合部区域中心城市和川东北经济区核心增长极，实现由工业大市向工业强市、经济大市向经济强市、总体小康向全面小康“三个跨越”。

根据上述总体要求，实现“十三五”规划目标，必须坚定走好符合达州实际的创新、协调、绿色、开放、共享发展之路。重点把握好以下五个方面：

——坚持创新发展，培育经济增长新动力。大力实施创新驱动战略，加快推进 “互联网+”行动计划，突出抓好供给侧结构性改革，深入实施传统产业升级、新兴产业培育、现代服务业提升、特色旅游品牌创建、科技创新“3215”【8】、大众创业万众创新“六大工程”，打造达州产业升级版。以“达推方案”为抓手，加快“四纵五横”铁路网【9】、“一环三纵五横”高速公路网【10】、域内快速通道建设，改造提升“六横六纵六联络”国省干线【11】，大力实施“3+10+1”骨干水利工程，建成达州新机场等一批标志性工程，夯实现代基础设施体系。深入推进6大重点领域和22个关键环节改革，加快形成有利于创新发展的体制机制。

——坚持协调发展，形成平衡发展新格局。推动区域协调发展，支持“三区”借力主城优势加快新型工业化进程和现代服务业发展，引导5个县（市）发挥比较优势竞相发展，崛起一批经济强县、经济强镇。推进城乡统筹发展，着力打造宜居宜业宜商中心城区，同步推进县城、小城镇、幸福美丽新村建设，完善“四层架构”现代新型城镇体系。推动物质文明和精神文明协调发展，坚持以人民为中心的工作导向，用中国梦和社会主义核心价值观、红军精神凝聚全市改革发展共识。

——坚持绿色发展，构筑生态文明新家园。树立生态文明理念，推动城镇绿色发展，支持绿色清洁生产，引导和推动全社会形成绿色消费自觉。严格执行主体功能区发展定位，加强重点生态工程建设和生物多样性保护。积极发展绿色低碳循环经济，加强资源节约和循环高效利用，全面推进绿色建筑发展模式，构筑绿色经济体系。大力改善环境质量，深入实施“净空、净水、净土、净噪”行动，加强污染物综合防治和环境治理。创建国家森林城市。开展绿化达州行动，加强江河流域治理，全面建成嘉陵江上游生态屏障。

——坚持开放发展，开创合作共赢新局面。积极对接国家内陆、沿江、沿边开放战略布局，完善全方位开放合作体系，形成横贯东西、连接南北的对外经济走廊，建设国家开放口岸和四川对外开放新高地。深化全方位开放合作，全面融入成渝发展，加快构建与万州港陆水联运体系，主动参与“一带一路”、长江经济带建设。创新开放合作体制机制，打造投资促进平台，办好各类投资促进活动，加大招商引资力度，助力全市经济转型升级。

——坚持共享发展，顺应人民生活新期待。坚决打赢脱贫攻坚战，确保49.85万贫困人口全部脱贫、7个贫困县全部摘帽，建成全省脱贫攻坚示范区。推进基本公共服务均等化，加强社会保障体系建设，健全农村留守儿童和妇女、老人关爱服务体系。统筹各类教育改革发展，实现义务教育均衡化，促进教育公平和现代化发展。推进健康达州建设，健全覆盖城乡居民的基本医疗卫生制度，推广全民健身运动，增强人民体质。落实人口政策，积极应对人口老龄化，促进人口均衡发展。

各位代表，“十三五”规划目标任务，既做到了与全面建成小康目标相衔接，又充分考虑了未来五年发展的条件和趋势，是一个客观积极的目标，符合经济发展规律和达州发展实际。我们坚信，只要全市上下紧扣目标、奋勇争先，“十三五”规划纲要确定的各项目标任务一定能够全面完成！同步全面建成小康社会的目标一定能够如期实现！

三、2016年工作安排

2016年，是“十三五”开局之年，也是推进结构性改革的攻坚之年。我们将认真贯彻落实中央、省市系列决策部署，围绕“十三五”发展目标，坚持 “创新发展、保位争先、加快转型、脱贫攻坚”工作基调，以抓铁有痕、踏石留印的作风，扎实做好各项工作，为“十三五”发展开好局、起好步。全年经济社会发展的主要预期目标是：地区生产总值增长7.5%，全社会固定资产投资增长10%，社会消费品零售总额增长12%，地方一般公共预算收入增长7.5%，城乡居民人均可支配收入分别增长8.5%、9.5%，城镇登记失业率控制在4.1%以内，居民消费价格指数调控目标与全省一致，完成省政府下达的节能减排和环保任务。

围绕上述目标，重点抓好十项工作：

（一）全力稳定经济增长。充分发挥投资对稳增长的关键性作用和消费的基础性作用，着力打好政策“组合拳”，确保经济平稳运行。

扎实推进项目建设。突出抓好全市重大、重点工程项目建设，力争全年完成投资1460亿元以上。切实抓好基础设施建设，开工建设新机场、土溪口水库等项目，加快达营高速公路、达宣和达开快速通道、6座在建中型水库等项目进度，确保南大梁高速公路、农村电网改造等项目竣工投运，积极做好渠江航道升级改造、西达渝和成南达高铁、固军水库、达渠快速通道等项目前期工作。努力提升产业投资比重，全力推进新材料、智能装备、精细化工、现代物流等项目建设，加快形成新的增长点。对接国家、省上“十三五”专项规划编制和“补短板、调结构”重大工程包，谋划储备一批新的基础设施、重大产业、生态环保和民生社会事业项目，全力争取国家专项建设基金和更多项目进入规划。大力推广PPP合作机制，鼓励民间资本参与各类项目建设。

积极扩大消费需求。适应消费升级趋势，破除政策障碍，优化消费环境，维护消费者权益。支持发展养老、健康、家政、教育培训、文化体育等服务消费。壮大网络信息、智能家居、个性时尚等新兴消费。大力发展电子商务，加快国家和省级电子商务示范县、示范基地项目建设，鼓励线上线下互动，推动实体商业创新转型。积极参加全省“三大”市场拓展活动，精心办好各类节会促销，提升“达州造”产品市场占有率。落实带薪休假制度，规范旅游市场秩序，迎接正在兴起的大众旅游时代。

支持实体经济发展。着力改善市场预期，营造宽松环境，提高企业投资信心，支持各种所有制企业创新发展。着力抓好国家和省相关政策的对接争取，帮助困难企业走出困境。充分发挥财政、金融和产业政策协同支持效应，做优“税金贷”产品，扩大“园保贷”试点，新增信贷投放150亿元。加快实施土地整理、“双挂钩”项目，积极争取用地指标，统筹调度煤电油气运等生产要素，保障实体经济发展需求。

（二）加快推进产业转型升级。坚持调整存量与做优增量并举，推动传统优势产业与战略性新兴产业融合发展，加快构建现代产业体系。

推进工业转型发展。加快冶金、建材、化工、食品、纺织、医药等传统产业升级改造，支持企业应用新技术、新工艺、新材料，加快产品升级换代，完成技改投资260亿元。开工建设正原微玻纤等30个项目，竣工投产三聚氰胺等40个项目，加快新达泵业迁建等35个项目，力争全年升规入统企业达到50户，规模以上工业增加值增长7.2%。实施中国制造2025达州行动，加快发展工业机器人、高端工模具等装备制造产业和液晶显示器、光电光学薄膜等电子信息产业，积极发展建筑节能、高分子化工等新材料产业。实施“互联网+制造”，加快“两化”深度融合，推进工业智能化改造。

推进三产提质增量。优先发展现代物流、现代金融等生产性服务业，着力推进秦巴物流园区、马踏洞金融集聚区等项目建设。创新发展商贸、旅游、康养等生活性服务业，支持达川杨柳、通川复兴、宣汉北城等商贸集聚区建设，着力打造红星美凯龙、仁和春天、罗浮新城市广场等城市综合体。加强重点景区建设，启动巴山大峡谷、铁山森林公园等旅游综合开发，加快龙潭河养生康养基地、城郊都市农业体验区等重点项目建设，提升八台山、賨人谷、莲花世界等景区接待能力，办好四川省第五届温泉旅游节、四川省第七届乡村文化旅游节（夏季）暨开江第三届荷花节、大巴山第二届山地休闲旅游节等活动。服务业增加值增长10%。

推进农业发展方式转变。加强农田水利基础设施、农建综合示范区建设，提升农业综合生产能力。抓好现代农业、林业、畜牧业重点县建设，推进国家、省级现代农业示范园区建设，加快健康水产业发展。大力实施“一牌三化”和农业“4+8”工程，新发展特色产业基地10万亩，建设优质腊梅培育供应观赏产业园，新改建畜禽标准化规模养殖场100个，创建“三品一标”认证品牌30个。大力发展道地中药材和农副产品加工业，延伸产业链条，建设全国富硒优质农产品生产加工基地。积极培育农业新型经营主体，新增市级以上龙头企业10家、标准化农民专业合作社20家、市级示范家庭农场30家。加强农业科技创新，健全农业社会化服务体系。做好第三次全国农业普查。

（三）切实加强城乡建设管理。尊重城市发展规律，贯彻新型城镇化理念，实施新型城镇化规划，加强城市基础设施建设，着力改善城乡发展环境。

加快推进新型城镇化。大力实施主城带动战略，加快北城滨江、三里坪、马踏洞、莲花湖、长田坝新区建设，抓好塔沱片区、火车站片区等棚户区改造，加强城市景观规划建设，有序推进地下综合管廊和海绵城市建设。支持大竹开展“宜居县城建设行动”试点，宣汉、开江建设卫星城市，渠县建设Ⅰ型小城市，万源创建绿色城市。协同推进小城镇和幸福美丽新村建设，加快建设一批县城副中心和中心镇，新建幸福美丽新村300个。深化扩权强县、扩权强镇改革，支持大竹、渠县撤县设市和符合条件的乡改镇、镇改街道办事处。深化户籍制度改革，加快推进农业转移人口市民化，力争户籍人口城镇化率达32.4%、常住人口城镇化率提高1.4个百分点。

全力抓好缓堵保畅。加快环城路二期、金南大桥、凤凰山隧道等项目建设，扎实做好城市轨道交通、绕城高速公路前期工作，推动城市各组团交通迅速转换。打通滨河路北延线二期及城区断头路，建成塔石路改造一期、和谐家苑二期周边道路等，新建停车泊位1万个，切实减轻城区交通“堵头”。大力发展公共交通，优化公交线路设置，建成肖公庙、徐家坝公交首末站和徐家坝客运站。规范出租车运营管理，强力整治非法营运行为。实施精细化交通管理，加强智能交通系统建设，督促驾驶员文明行驶，引导市民文明、绿色出行，多措并举缓解城市交通拥堵。

深入实施“五治”工程。推进城市执法体制改革和管理重心下移，强化辖区政府、街道办事处管理职能，充分发挥社区基础作用，广泛发动市民共同参与管理。全面开展清洁城市环境活动，推广市场化保洁，提升清扫保洁机械化作业率。重拳整治“摊点乱摆、广告乱设、车辆乱停、行人乱穿”。强力整治扬尘污染，大力开展“大气污染防治”、“百日总量减排”攻坚计划，推动城区空气质量持续好转。强化噪声污染控制，建设安静舒适的城乡环境。加快中心城区第二污水处理厂、截污干管、乡镇污水处理厂等设施建设，不断改善流经城镇河流水质，确保饮用水源安全。

（四）继续深化改革创新。围绕解决重点领域的突出矛盾和问题，加快破除体制机制障碍，大力实施创新驱动发展战略，进一步激发市场活力和社会创造力。

深化重点改革。推动简政放权、放管结合、优化服务改革向纵深发展，继续取消和下放行政审批事项，完善权力、责任、负面“三张清单”管理制度。加快新一轮国企分类改革，大力发展混合所有制经济，推动达商集团战略重组和国有粮食企业改革，组建城市公交集团和文化旅游产业投资公司。深化商事制度改革，新增民营市场经营主体1.1万个。完善分税制财政体制和政府预算体系，转变财政支持发展方式，构建产业发展投资引导基金体系和财政金融互动政策体系。继续推进农业农村“6+10”专项改革，基本完成“六权同确”【12】，实现不动产统一登记。抓好省上专项改革方案对接，推动各项重大改革落地见效。

强化创新驱动。深化科技体制改革，鼓励企业技术创新、产品创新、运营模式创新，新增高新技术企业3家，新培育重点实验室、企业技术中心等创新平台10家，申请专利900件。支持创建微玻纤产业技术应用研究院。实施“双创”达州行动，办好“双创周”、“创青春”等活动，抓好达商、农民工返乡创业和青年创新创业工作。加强各类人才引进，推动海内外达州籍人才回乡发展。支持行业领军企业、创业投资机构、社会组织积极参与或自主建设众创空间，激发全社会创新创造活力。

（五）加强供给侧结构性改革。推进供给侧结构性改革，是适应和引领经济发展新常态的必然要求。我们将抓住关键环节，用结构性改革的办法破解发展难题。

稳妥化解过剩产能。科学确定化解过剩产能目标任务，制定具体实施方案和配套措施。积极争取国家和省上政策，支持钢铁、煤炭、水泥等产能过剩行业兼并重组、转型升级。严格把关新发展新引进项目，坚决防止新增过剩产能。积极稳妥处置“僵尸企业”，分类有序实施，同步做好职工安置工作。

积极化解房地产库存。提高棚改货币化安置比例，深化购租并举的城镇住房制度改革。大力发展住房租赁市场。认真落实房地产新政，发挥住房公积金、银行按揭贷款、购房财政补贴等作用，提高群众住房消费能力。科学调控土地出让及房地产开发量，积极培育本地建筑企业，促进房地产业健康发展。

有效防范金融风险。依法解决民间借贷和非法集资问题，健全担保体系，完善金融监管机制，坚决守住不发生系统性和区域性金融风险的底线。发挥好企业应急转贷资金作用，防范企业资金链断裂风险。加强政府性债务监测监管，规范举债行为，积极争取国家债务置换，防范和化解地方政府性债务风险。

切实减轻企业负担。认真落实国家和省降低实体经济企业成本政策，清理规范中介服务收费，坚决遏制涉企乱收费行为。积极争取和用好直购电，推动富余电消纳，扩大直供气规模。采取租赁、租让结合等多种供地方式，降低企业用地成本。支持发展多式联运、农产品冷链物流，降低企业物流成本。清理银行业金融机构不合理收费，提高直接融资比重，降低企业融资成本。

（六）全面扩大开放合作。坚持把扩大开放作为后发赶超的战略支撑，着力打造全省对外开放新高地。

深化区域合作。发挥承东启西、连南贯北的区位优势，加快构建综合立体交通走廊，主动参与“一带一路”和长江经济带建设，积极融入对外开放大格局。全面融入成渝发展，加快建设成渝经济区配套产业高地。扩大与东部地区、沿海地区的交流合作，构建产业转移“桥头堡”。加强与川东北、鄂西、陕南片区的交流合作，推动区域基础设施互联互通，促进共同发展。

提升开放实效。继续推行市领导牵头10亿元以上招商项目制度，加快已签约重点项目落地实施，用好西博会、渝洽会等省内外大型展会平台，办好“知名企业达州行”等投资促进活动，力争引进一批高端型、科技型知名企业，全年招商引资到位资金650亿元。搭建对外开放平台，争取设立达州海关，支持达州经开区创建省级高新区。建好达州经开区、大竹工业园区外向型产业基地，鼓励企业充分开拓和利用国际国内两个市场，大力开展对外贸易、工程承包、劳务和技术合作、境外投资，力争进出口总额增长9%以上。

（七）扎实推进脱贫攻坚。坚持把脱贫攻坚作为第一民生工程来抓，突出打好“3+10”组合拳【13】，确保全年15万人脱贫、149个建档立卡贫困村“摘帽”。

夯实脱贫攻坚工作基础。编制完成“十三五”扶贫总体规划和专项规划，制定实施10个专项扶贫方案年度计划。认真开展贫困人口信息数据对比、排查核实，全面完成贫困人口规模、致贫原因、扶持举措等信息入库。积极开展贫困统计监测，加强贫困动态管理，努力提高扶贫质量。

扎实推进精准脱贫工程。把“六个精准”【14】、“六个一批”【15】要求真正落实到村、到户、到人。抓好基础设施、产业发展、医疗卫生、易地搬迁等十大扶贫工程，新改建农村公路2100公里，新解决20万农村人口饮水安全问题，新建农村廉租住房2735套、改造农村危房1.5万户，易地扶贫搬迁2.9万人。大力推进旅游扶贫、电商扶贫、科技扶贫，切实增加贫困群众收入。统筹解决好插花式贫困问题。

着力创新脱贫攻坚机制。认真执行脱贫攻坚目标、任务、资金、权责“四到县”制度，持续增加市县财政投入，整合行业涉农资金，保障重点项目实施。扎实开展驻村帮扶，深化市级部门对口帮扶。鼓励民主党派、群团组织、企业高校、科研院所，采取多种形式参与扶贫，形成脱贫攻坚的强大合力。构建扶贫资金多元投入机制，引导更多金融资金和社会资本参与扶贫开发。

（八）强化生态环境保护。良好生态环境是最普惠的民生福祉。我们将坚定不移走绿色发展之路，让青山绿水长留、蓝天白云常驻！

开展绿化达州行动。严格划定和控制生态保护红线，统筹推进城乡绿化美化，突出抓好“沿路、沿河、沿库”生态廊道建设，切实加强自然保护区、森林公园、地质公园、湿地公园保护建设。启动创建国家森林城市。继续抓好天保二期工程、新一轮退耕还林建设，完成营造林20万亩，新增森林5000公顷。加强地质灾害防治，治理水土流失100平方公里。

全面推进节能减排。大力发展绿色经济，推进工业、建筑、交通等重点领域节能降耗，支持采购绿色环保产品，扩大节能产品运用。加快“气化达州”步伐。加强生活垃圾分类回收和再生资源回收利用，开展能源和水资源消耗、建设用地、碳排放等总量和强度双控行动。实施循环发展引领计划，推进“1+7”园区循环化改造试点示范，引导企业循环式生产。大力开展大气、水、土壤污染防治行动，推进工业污染源、机动车尾气综合整治，加强农村面源污染和畜禽养殖污染治理，强化重金属污染防控。加强环境监测、执法监管及其能力建设，加大执法惩戒力度。

（九）持续加强民生社会建设。民之所望，即施政所向。我们将切实加大民生和社会事业投入，深化幸福达州、平安达州、法治达州建设，让改革发展成果更多惠及全市人民。

切实保障和改善民生。实施“九项民生工程”，办好18件民生大事，确保全市一般公共预算民生支出比例不低于70%。坚持以创业带动就业，突出抓好高校毕业生、农民工、贫困家庭人员等重点人群就业和再就业，新增城镇就业3.4万人。实施全民参保计划，推进机关事业单位养老保险制度改革。加强养老服务体系建设，新增（改造）养老床位4900张，新建城乡社区日间照料中心110个。加快城乡居民医保整合，推进医保市级统筹，实现大病保险全覆盖。统筹推进社会救助，提高城乡低保水平，发展慈善事业，深化“量体裁衣”式残疾人服务。切实保障妇女、儿童权益，加强对农村留守儿童和妇女、老人的关爱服务。

大力发展社会事业。推进义务教育均衡发展，新改扩建中小学校100所、公办幼儿园26所、特殊教育学校1所，确保达州嘉祥外国语学校、通川七小新锦校区和一小莲湖校区、达川实验小学杨柳校区等建成招生。逐步提高高中阶段教育普及水平；加快建设西南职教园区，完成达州职业技术学院主体搬迁；支持四川文理学院发展建设，提升教学水平和创新能力；推动继续教育、特殊教育等改革发展。实施“健康达州”建设，完善分级诊疗制度，引导优质医疗资源下沉，加强重大传染病综合防治，促进中医药事业发展。实施文化惠民工程，繁荣文艺创作，加强巴文化遗址保护利用，办好第六届全国新农村文化艺术展演活动。加快数字档案馆、群团组织社会服务中心建设。开展全民健身活动，办好市第三届运动会暨残疾人运动会。落实“两孩”政策，开展应对人口老龄化行动。继续加强民族、宗教、对台、外事、侨务、统计、保密、地方志等工作。

提高社会治理水平。坚持预防为主，注重源头治理，强化应急防范和处置，有效化解各类矛盾和问题。开展信访“四无”【16】创建活动，实行网上受理信访制度，有效化解信访积案。加强人民调解组织建设。健全基层依法治理体系，加强社区、社会组织、社会工作者“三社联动”，支持群团组织参与社会治理。启动“七五”普法，深化“法律七进”。加强天网工程、立体化社会治安防控体系建设，依法严厉打击各类违法犯罪活动。切实抓好公共安全监管，加大“禁毒防艾”工作力度。持续推进“打非治违”和安全专项整治，坚决遏制重特大事故发生。严格食品药品安全监管，保障群众饮食用药安全。做好地震、测绘、气象、水文工作，有效防范和应对重大自然灾害。

大力支持驻达解放军和武警部队建设，切实加强国防教育、国防动员、人民防空、交通战备、民兵预备役、双拥共建等工作。

（十）努力建设人民满意政府。美好的发展愿景，人民的殷切期盼，对政府工作提出了新的更高要求。我们将聚焦依法行政、廉洁勤政，切实加强法治政府和服务型政府建设。

坚持依法履职。严格遵守宪法和法律，坚持法定职责必须为、法无授权不可为，把依法行政的要求贯穿于政府决策、执行、落实的全过程。深化行政执法体制改革。加强行政复议和行政应诉工作。深入推进政务公开。主动接受市人大及其常委会的法律监督和工作监督，自觉接受市政协的民主监督以及社会和舆论监督，让权力在阳光下运行。

坚持廉洁履职。认真执行《廉洁自律准则》，严格落实党风廉政建设责任制，努力使清正廉洁成为每一个政府工作人员的行动自觉。坚持把纪律和规矩挺在前面，加强行政监察、审计监督，推动正风肃纪常态化。以减权限权、创新监管等举措减少权力寻租空间，铲除滋生腐败土壤。推动廉政建设向基层延伸，坚决纠正侵害群众利益的不正之风，坚定不移惩治腐败。

坚持勤勉履职。深入践行“三严三实”，认真抓好“两学一做”【17】学习教育，切实增强政治意识、大局意识、核心意识、看齐意识，着力打造高素质公务员队伍。健全并严格执行工作责任制，确保各项决策部署全面落实、政策措施全面落地。健全督查问责机制，坚决整治庸政懒政怠政行为。健全激励机制，让广大干部愿干事、敢干事、干成事。

各位代表，达州发展已站在新的历史起点上，让我们紧密团结在以习近平同志为总书记的党中央周围，在省委、省政府和市委的坚强领导下，紧紧依靠全市人民，坚定信心、鼓足干劲、开拓创新、扎实工作，为实现“十三五”宏伟蓝图、同步全面建成小康社会而努力奋斗！
